# Contrastive Analysis of the Raman Spectra of Polychlorinated Benzene: Hexachlorobenzene and Benzene

**DOI:** 10.3390/s111211510

**Published:** 2011-12-08

**Authors:** Xian Zhang, Qin Zhou, Yu Huang, Zhengcao Li, Zhengjun Zhang

**Affiliations:** 1 Advanced Materials Laboratory, Department of Materials Science and Engineering, Tsinghua University, Beijing 100084, China; E-Mails: xian-zhang09@mails.tsinghua.edu.cn (X.Z.); qinzhou@tsinghua.edu.cn (Q.Z.); huang-y07@mails.tsinghua.edu.cn (Y.H.); zcli@tsinghua.edu.cn (Z.L.); 2 Institute of Nuclear and New Energy Technology, Tsinghua University, Beijing 100084, China

**Keywords:** persistent pollutants, polychlorinated benzene, homologue, isomer, hexachlorobenzene, vibration mode

## Abstract

Detection of persistent pollutants such as polychlorinated benzene in environment in trace amounts is challenging, but important. It is more difficult to distinguish homologues and isomers of organic pollutantd when present in trace amounts because of their similar physical and chemical properties. In this work we simulate the Raman spectra of hexachlorobenzene and benzene, and figure out the vibration mode of each main peak. The effect on the Raman spectrum of changing substituents from H to Cl is analyzed to reveal the relations between the Raman spectra of homologues and isomers of polychlorinated benzene, which should be helpful for distinguishing one kind of polychlorinated benzene from its homologues and isomers by surface enhanced Raman scattering.

## 1. Introduction

Persistent organic pollutants (POPs), such as dioxins, polychlorinated biphenyls (PCBs) and polychlorinated benzene *etc.*, are harmful to humans’ health, and have seriously polluted the environment [1]. In recent years, great research interest has been focused on the detection and removal of these pollutants, in which techniques that are able to detect these compounds in trace amounts are essential. This is because they can accumulate to a high concentration in human bodies through the food chain and cause severe diseases when the concentration exceeds the critical level, even when just just in trace amounts in the environment [1–3]. Currently, the combination of high-resolution gas chromatography (GC) and mass spectrometry (MS) is widely used as a powerful means for the detection of these pollutants. However, the GC/MS method is expensive and time-consuming, and is not able to distinguish homologues [4–7].

Nanostructured materials exhibit many interesting properties and may have potential in the detection of trace amounts of organic pollutants [8–11]. For example, some organics were detected at trace levels by Surface-Enhanced Raman Scattering (SERS), using nanostructures of noble metals (Cu, Ag and Au) as the substrate [12–18]. SERS is a technique with high sensitivity, simplicity and fast detection, and it’s also capable in the recognition of compounds.

**Figure 1.**
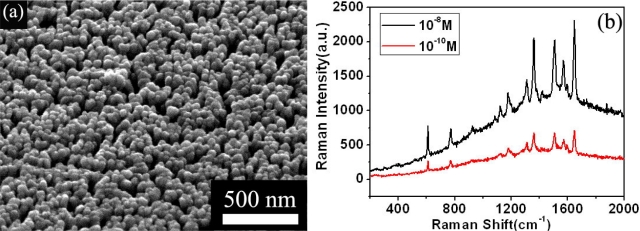
**(a)** The SEM image of vertically aligned Ag nanorods deposited on silicon substrates; and **(b)** Raman spectra of R6G on these Ag nanorods as the SERS substrate, at a concentration of 10^−8^ M and 10^−10^ M, respectively.

Among the approaches so far available to fabricate SERS-active substrates, glancing angle deposition (GLAD) is a promising one because of its simplicity and effectiveness [19]. Porous films consisting of Ag isolated nanorods is obtained through GLAD, and found to be excellent SERS substrates for the detection of some organics, with a SERS enhancement of greater than 10^12^. The structure and SERS performance of porous Ag film is shown in Figure 1(a,b), respectively.

Although the SERS technique could be used to detect trace amounts of polychlorinated benzenes, distinguishing one kind of polychlorinated benzene from its 12 isomers and homologues is still a problem. Indeed, isomers and homologues of organic pollutants have similar physical and chemical properties, thus, they are hard to distinguish, especially in trace amounts. As different isomers and homologues have different vibration modes, their Raman shift corresponding to these vibration mode are different, too. By this way, the SERS method with silver nanorods as a substrate can be used to identify the Raman characteristics of homologues and isomers of polychlorinated benzene, even at trace levels, so it is necessary to analyze the differences and relationships between the Raman spectra of each kind of polychlorinated benzene if we want to distinguish each homologue or isomer. As a tentative research topic, we analyzed the Raman spectra of hexachlorobenzene and benzene with their corresponding vibration modes. Here, we report our theoretical study on the Raman spectra of hexachlorobenzene and benzene.

## 2. Experimental Procedure

The Raman spectra of benzene and hexachlorobenzene (HCB) were measured with a Renishaw Raman 100 spectrometer using a 633 nm He-Ne laser as the excitation source at room temperature. Powders of these compounds are commercially available from the AccuStandard Company. Simulation of these Raman spectra was performed using the Gaussian 03 programme package with the density functional theory, to better understand the vibrational modes observed and figure out fingerprints of these compounds. The simulations were carried out with the Becke’s three-parameter hybrid method using the Lee-yang-Parr correlation functional (B3LYP) and the LANL2DZ basis set [20]. The B3LYP functional has been employed with success to simulate the Raman spectra of organic pollutants [21]. The Gaussian View was used to input investigated compounds’ data visually.

## 3. Results and Discussion

**Figure 2.**
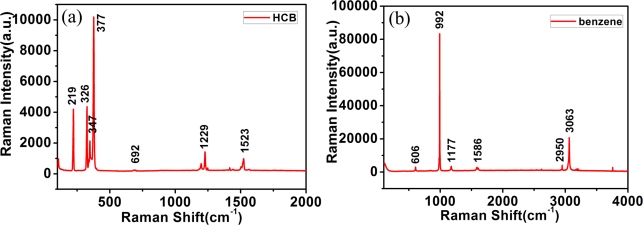
Experimental Raman spectrum of **(a)** hexachlorobenzene; **(b)** benzene, in powder, with the main peaks marked.

First of all, the standard Raman spectra of hexachlorobenzene and benzene in the powder state is recorded by the Renishaw Raman spectrometer using a 633 nm laser as excitation source. Figure 2(a,b) shows the measured Raman spectra of hexachlorobenzene (HCB) and benzene, respectively. Each material has strong peaks at ∼1,500 cm^−1^, 1,000 cm^−1^, 650 cm^−1^, demonstrating the common features of benzene and hexachlorobenzen, but the differences between the Raman spectra of benzene and hexachlorobenzene are obvious. For example: (1) hexachlorobenzene has strong peaks at 377 cm^−1^, 347 cm^−1^, 326 cm^−1^, 219 cm^−1^; (2) benzene has strong peaks at 3,063 cm^−1^, 2,950 cm^−1^, 1,177 cm^−1^. To gain a clear understanding of these features, we performed a simulation using the Gaussian 03 programme package.

**Figure 3.**
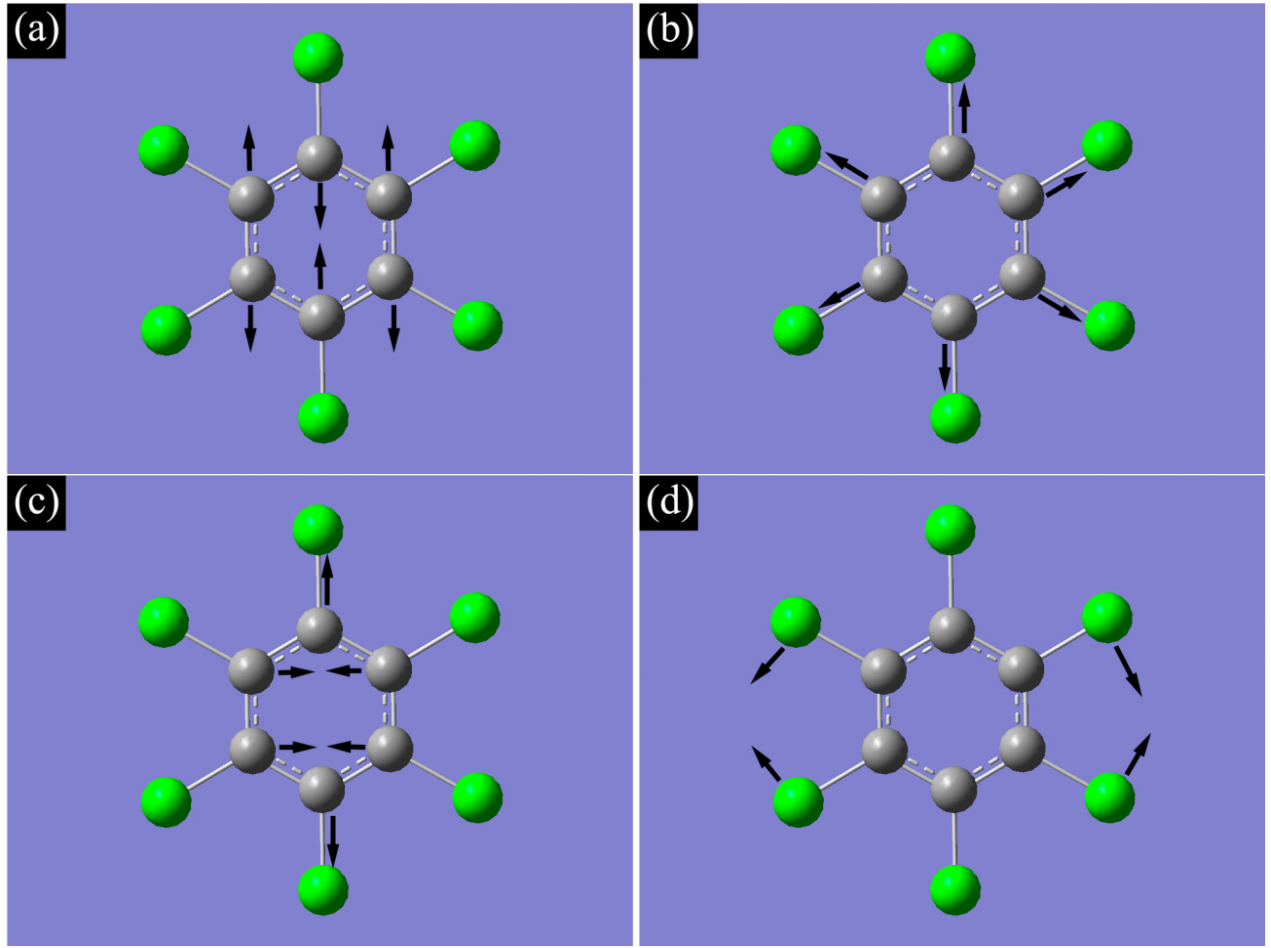
Illustration of vibration in hexachlorobenzene for: **(a)** CCC stretching vibration mode; **(b)** benzene ring breathing vibration mode; **(c)** CCC deformation in plane vibration mode; **(d)** C-Cl shear vibration mode.

From Figure 2(a), one clearly sees peaks at ∼1,523, 1,229, 692, 377, 347, 326 and 219 cm^−1^, demonstrating the feature of HCB. It is found by simulation that the peak at 1,523 cm^−1^ represents the CCC stretching vibration mode, shown as Figure 3(a); the peak at 1,229 cm^−1^ represents the benzene ring breathing vibration mode, shown as Figure 3(b); the peak at 692 cm^−1^ represents the CCC deformation in plane vibration mode, shown as Figure 3(c); the peak at 377 cm^−1^ represents the C-Cl symmetrical stretching vibration mode; the peak at 347 cm^−1^ represents the benzene ring swing out-of-plane vibration mode; the peak at 326 cm^−1^ represents the C-Cl anti-symmetrical stretching vibration mode; and the peak at 219 cm^−1^ represents the C-Cl shear vibration mode, shown as Figure 3(d).

Figure 2(b) shows the measured Raman spectrum of benzene, where the peaks at 3,063, 2,950, 1,586, 1,177, 992 and 606 cm^−1^ represent its characteristic features. It is found by simulation that the peak at 3,063 cm^−1^ represents the C-H symmetrical stretching vibration mode; the peak at 2,950 cm^−1^ represents C-H anti-symmetrical stretching vibration mode; the peak at 1,586 cm^−1^ represents the CCC stretching vibration mode; the peak at 1,177 cm^−1^ represents the C-H shear vibration mode; the peak at 992 cm^−1^ represents the benzene ring breathing vibration mode; the peak at 606 cm^−1^ represented the CCC deformation in plane vibration mode.

By contrasting the Raman spectra of HCB and benzene, it is found that the vibrational Raman shifts just involving C atoms are stable whether the substituent atom is Cl or H. For example, the Raman shifts of the CCC stretching vibration modes in HCB and benzene are 1,523 cm^−1^ and 1,586 cm^−1^, respectively. The Raman shifts of the benzene ring breathing vibration mode in HCB and benzene are 1,229 cm^−1^ and 992 cm^−1^, respectively. The Raman shifts of CCC deformation in plane vibration mode in HCB and benzene are 692 cm^−1^ and 606 cm^−1^, respectively, so recognizing the peaks around 1,500 cm^−1^, 1,000 cm^−1^ and 650 cm^−1^ could be an effective way to detect the homologues and isomers of polychlorinated benzene. However, the Raman shift of vibration involving substituent atoms depends on the substituent. For example, the C-Cl symmetrical stretching vibration mode Raman shift in HCB is at 377 cm^−1^, while the Raman shift of the C-H symmetrical stretching vibration mode in benzene is at 3,063 cm^−1^. The Raman shift of the C-Cl anti-symmetrical stretching in HCB is 326 cm^−1^, while the Raman shift of the C-H anti-symmetical stretching in benzene is 2,950 cm^−1^. The Raman shift of C-Cl shear vibration mode is at 219 cm^−1^, while the Raman shift of C-H shear vibration mode is at 1,177 cm^−1^. The Raman shifts of the same kind of vibration involving substituents vary in HCB and benzene, and the Raman shifts become smaller as the substituent atom changes from H to Cl, so the Raman shift of vibration involving the substituent atom can help us distinguish one kind of chlorinated benzene from its homologues and isomers.

Additionally it is found for hexachlorobenzene that the Raman shift of vibration involving just C atoms is around 500–2,000 cm^−1^, while the Raman shift of vibration involving Cl substituent atoms is around 100–400 cm^−1^. Subsequently, the two kinds of vibrations correspond to Raman peaks separated into two bands in hexachlorobenzene, while the Raman peaks of the two kinds of vibration mix into one band in benzene.

## 4. Concluding Remarks

In summary, we demonstrated here an analysis of the Raman spectra and the corresponding vibration modes of hexachlorobenzene and benzene. It is shown that the simulation and analysis of Raman spectra can be a powerful way to detect homologues and isomers of polychlorinated benzene in trace amounts using the SERS technique, which is crucial for the removal of the pollutants.
